# Multi-omics analysis reveals tissue-specific biosynthesis and accumulation of diterpene alkaloids in *Aconitum japonicum*

**DOI:** 10.1007/s11418-025-01881-y

**Published:** 2025-03-20

**Authors:** Megha Rai, Amit Rai, Tetsuya Mori, Ryo Nakabayashi, Michimi Nakamura, Marsheige Kojoma, Hideyuki Suzuki, Kazuki Saito, Mami Yamazaki

**Affiliations:** 1 Graduate School of Pharmaceutical Sciences, Chiba University, Chiba, Japan; 2 Crop Sciences, University of Illinois Urbana Champaign, Illinois, USA; 3 RIKEN Center for Sustainable Resource Science, Yokohama, Japan; 4 Faculty of Pharmaceutical Sciences, Health Sciences University of Hokkaido, Hokkaido, Japan; 5 Kazusa DNA Research Institute, Chiba, Japan; 6 Plant Molecular Science Center, Chiba University, Chiba, Japan

**Keywords:** Diterpenoids, Aconitine, Multi-omics, Traditional medicinal plants, Metabolomics

## Abstract

*Aconitum japonicum*, native to the mountainous regions of Japan, is a toxic perennial plant widely recognized for its therapeutic potential. Despite its pharmacological importance, the complete biosynthetic pathway of diterpene alkaloids, bioactive compounds with significant pharmaceutical implications and derived from *Aconitum* species, remains elusive. In this study, leveraging high-throughput metabolome and transcriptome analyses, we conducted a comprehensive investigation using four tissues of *A. japonicum*, including leaf, mother root, daughter root, and rootlet. By integrating these multi-omics datasets, we achieved a holistic insight into the gene expression patterns and metabolite profiles intricately linked with diterpene alkaloid biosynthesis. Our findings unveil potential regulatory networks and pinpoint key candidate genes pivotal in diterpene alkaloid synthesis. Through comparative analyses across tissues, we delineate tissue-specific variations in gene expression and metabolite accumulation, shedding light on the spatial regulation of these biosynthetic pathways within the plant. Furthermore, this study contributes to a deeper understanding of the molecular mechanisms dictating the production of diterpene alkaloids in *A. japonicum*. Besides advancing our knowledge of plant secondary metabolism in *A. japonicum*, this study also provides a high-quality multi-omics resource for future studies aimed at functionally characterizing the target genes involved in different metabolic processes.

**Graphical abstract:**

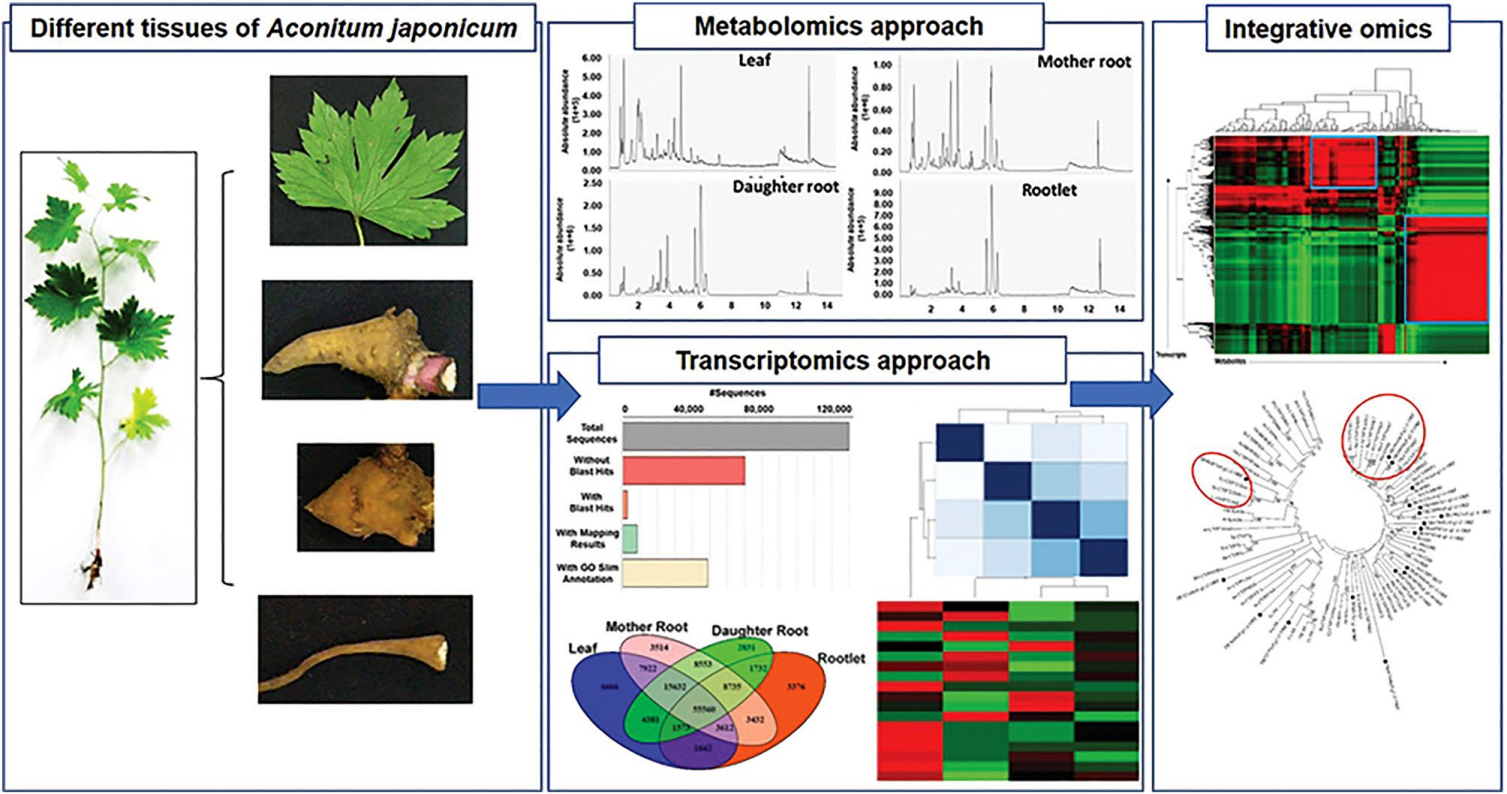

**Supplementary Information:**

The online version contains supplementary material available at 10.1007/s11418-025-01881-y.

## Introduction

*Aconitum japonicum* stands out among plant species of the diverse *Aconitum* genus for its pharmacological relevance being recognized in several traditional medicinal practices [1]. Among more than 350 known *Aconitum* species [2], only two, namely *A. japonicum* and *A. carmichaelii*, are listed in the Japanese Pharmacopoeia [3]. *A*. *japonicum*, also known as “Yamatorikabuto” in Japanese due to its inhabitation in the mountainous regions, is an inherent species of Japan distributed along the eastern Kanto district, Pacific Tohoku district, and subalpine zone of the Chubu district [4]. The rhizomes of *A. japonicum,* called “Udzu” or “Bushi” in Kampo medicines, have long been used for treating various medical conditions, including dysuria, neuralgia, gouts, hypometabolism, chills, cardiac weakness, several neuropathic pains, and rheumatic diseases [5]. Udzu is considered as one of the “Four Pillars of Medicine” by the ancient traditional medicinal systems [1, 6], and over 20 herbal formulations includes Fuzi as the main composition [7]. However, the associated neuro- and cardio-toxicity with the *Aconitum* species, and their misuse in suicide and homicide cases, makes it a double-edged sword that requires additional regulation for its commercial level farming and uses in medicinal practices [8].

Several studies have attributed the presence of bioactive metabolites to the substantial medicinal properties of *Aconitum* plants, including C_18_-, C_19_-, and C_20_-types diterpene alkaloids, mainly accumulated within the root-tissues [9]. The C_19_-type diterpene alkaloids, including aconitine, mesaconitine, jesaconitine, and hypaconitine, are the major alkaloid content of *A. japonicum*, which are regulated for medicinal use due to their acute toxicity [5, 10]*.* The biosynthetic pathway of the diterpene alkaloids in *Aconitum* species, except for the biosynthesis of its precursor molecule, atisine, remains largely unknown, [11]. The condensation of three molecules of isopentenyl pyrophosphate, derived through the mevalonate (MVA) and methylerythritol (MEP) pathway, and catalyzed by geranylgeranyl diphosphate synthase (GGPPS), results in the formation of geranylgeranyl pyrophosphate (GGPP) [11–13]. GGPP then undergoes bicyclization to form kaurene or atisene through alternate rearrangement reaction catalyzed by diterpene synthases, copalyl diphosphate synthase, and kaurene synthase, respectively. The ensuing kaurene and atisene core chemical structures are further oxidized and hydroxylated to form the atisine skeleton by the incorporation of a β-ethanolamine moiety derived from the decarboxylation of L-serine [14–16]. The atisine skeleton, thus formed, undergoes a series of modifications and rearrangements to derive the diversity of *Aconitum* diterpene alkaloids that imparts its known medicinal properties [17, 18]. The biosynthesis pathway responsible for the modifications of the atisine skeleton toward the characteristic diterpenoids found in *Aconitum* species, including the enzymes and metabolite intermediates involved, remains largely unknown.

In recent years, efforts have been made to characterize the pharmacological and toxicological characteristics of raw herbal formulations of *Aconitum* species to identify their major alkaloids and their derivatives [1, 5, 19, 20]. These studies have been developed to enable quality control and quality assurance for the intake of *Aconitum* rhizome as a drug [6, 21, 22]. However, the lack of metabolome and genome resources are major impediments to explore and achieve sustainable agricultural practices, and identification of molecular markers for selection of medicinally valuable *Aconitum* accessions. Only limited studies to date have been undertaken to establish the genetic resources and its association with metabolite constituents for these medicinally important *Aconitum* species [11, 15, 23–26]. While several important medicinal plants currently lack high-quality genome assembly, owing to their large genome size and polyploidy, RNA-seq based transcriptome assembly has been expanded to diverse genomes for establishing the genetic resources of non-model medicinal plant species [27, 28]. RNA-seq reads can be assembled de novo in the absence of genomic resources; however, the sequence-homology-based annotation for the assembled transcripts lead to multiple transcripts being assigned to a single enzymatic step [29]. Nevertheless, given the fact that a minimal set of core molecules, which are acted upon by enzyme families like transferases, oxidases, and hydroxylases, among others, are involved in the biosynthesis of specialized metabolites, co-expression-based integration of the transcriptome datasets and metabolome datasets have been established as a successful strategy for identification of novel functional genes [30–33].

Here, we established and describe high-quality metabolome and transcriptome resources for *A. japonicum* using leaf and three different root types, namely mother root (the main root), daughter root (the new roots that sprout from the main root), and rootlet (fibrous roots that help the plant in anchoring). Each of the four tissue types used in this study represents a unique reservoir of metabolites, contributing to the plant’s overall medicinal profile. We have used multi-omics analysis, employing both transcriptomics and metabolomics data integration, to understand the molecular machinery responsible for the synthesis and accumulation of bioactive diterpene alkaloids. Untargeted metabolome analysis and annotation revealed the distribution pattern of key diterpene alkaloids with known medicinal properties. The established de novo transcriptome assembly identified key active biological processes and their distinct associations across various tissues of *A. japonicum.* Further, co-expression-based integration of the transcriptome and metabolome datasets facilitated the identification of the known components, as well as proposing the unknown components involved in the biosynthesis of diterpene alkaloids in *A. japonicum.* Through our multi-omics approach, integrating transcriptomics and metabolomics data, we hereby provide a holistic understanding of the molecular landscape within these diverse tissues. This study is poised to unravel the genetic underpinnings, transcriptional regulations, and metabolite pathways that define the unique biochemical makeup of each tissue type. While transcriptomic and metabolomic datasets from model species are widely available, the transcriptome and metabolome resources generated for *A. japonicum* in this study fill a critical gap by offering insights into gene expression and metabolite profiles in medicinal plants, as has been reported earlier for *Mallotus japonicus* [32] and *Cornus officinalis* [33] among others. Moreover, the resources established in this study hold the potential to not only deepen our understanding of the medicinal properties of *A. japonicum* but also pave the way for targeted breeding and cultivation strategies aimed at enhancing the yield of bioactive compounds for medicinal applications.

## Materials and methods

### Plant materials

*A. japonicum* plants were maintained in the green house of Faculty of Pharmaceutical Sciences, Health Sciences University of Hokkaido, Japan. All four tissue types of *A. japonicum*, namely, leaf (LF), mother root (MR), daughter root (DR), and rootlet (RT), were harvested from five individual plants of *A. japonicum* as biological replicates on ice and snap frozen using liquid nitrogen before storing at -80 °C until samples were processed for RNA and metabolite extraction.

### Untargeted metabolite analysis for four tissues of *Aconitum japonicum* using high-resolution mass spectrometry approach (UPLC–QTOF-MS)

For metabolome analysis, five individual biological replicates for three tissues, namely MR, DR, and LF tissues, and two individual biological replicates for RT were collected for further analysis. The tissues of *A. japonicum* were freeze-dried using a freeze dryer (FDU-2200) (Tokyo Rikakikai CO., Ltd., Tokyo, Japan), and were subsequently used for the metabolite extraction. Metabolites were extracted using 50 μL of 80% (v/v) LC–MS-grade methanol (Wako chemicals, Osaka, Japan) and 20% (v/v) LC–MS-grade water (Wako chemicals, Osaka, Japan) containing 2.5 µM of 10-camphoursulfonic acid (TCI, Tokyo, Japan), and 2.5 μM of lidocaine (TCI, Tokyo, Japan) per milligram of dry weight. The mixture was homogenized using a mixer mill, MM300 (Retsch, Haan, Germany), with a zirconia bead at 18 Hz at 4 °C for 7 min. followed by centrifugation at 17,800 × g for 10 min. The supernatant was subsequently processed using Oasis HLB μElution plate (Waters Inc., Milford, MA, USA) for removal of lipids and impurities, and the MS and MS/MS datasets were acquired in the positive and negative ionization mode as described previously [32, 34]. The data were processed using MS-DIAL v4.80 [35] with default parameters, and the acquired metabolite peaks were used for Principal Component Analysis (PCA). The MS/MS-based validation of metabolite identity was performed using MS-FINDER v3.52, as described previously [32, 36]. The average level for the five bio replicates of MS/MS-validated metabolites was used further for the correlation-based integration of the transcriptome and metabolome datasets.

### RNA extraction and cDNA library preparation

Frozen tissues from single biological replicates of *A. japonicum* were used for RNA extraction following RNeasy Plant Mini Kit (Qiagen, USA) and cDNA library were prepared using SureSelect Strand Specific RNA library kit (Agilent Technology, USA), according to the manufacturer’s instruction. RNA extraction, RNA integrity analysis, mRNA sample preparation, fragmentation of isolated mRNA, and cDNA library preparation for Illumina sequencing were performed as described previously [37].

### Illumina sequencing

The cDNA libraries thus prepared for each tissue of *A. japonicum* was sequenced using an Illumina HiSeq™ 2000 sequencer (Illumina, Inc., San Diego, CA, USA), and paired-end reads were obtained with an average length of 101 bps. Preparation and shearing of mRNA, cDNA library preparation, and sequencing were performed at Kazusa DNA Research Institute, Chiba, Japan. The raw sequence reads for all four tissues of *A. japonicum*, their expression value and the de novo transcriptome assembly used in this study have been deposited in NCBI’s Gene Expression Omnibus (GEO) and are available at GEO series accession number GSE275867.

### RNA-seq raw reads pre-processing, de novo transcriptome assembly, and functional classification of transcripts

Raw sequencing reads, thus generated, were preprocessed through the Trimmomatic program v0.39 [38] to remove adaptor sequences, short reads, reads with ambiguous ‘N’ base > 5%, and low-quality reads (Phred score < 30). Paired-end processed reads, as well as unpaired high-quality reads that lost their corresponding sequence partner due to Trimmomatic filtering, for all four tissue types were combined to build a de novo transcriptome assembly of *A. japonicum*. The de novo transcriptome assembly resulting from the Trinity program v3.0 [39] was further processed through the CD-HIT-EST program v4.8.1 [40] for sequence redundancy removal, and subsequently used for annotation and characterization. The transcripts, thus, obtained were used for the read alignment and abundance estimation in individual tissues of *A. japonicum* using Bowtie 2.0 v2.3.5.1 [41] and RSEM v1.3 [42], respectively. Transcript expression was calculated in terms of Fragments per Kilobase exon per million mapped fragments (FPKM). Correlation analysis was performed for all four tissues of *A. japonicum* through the DESeq2 program v3.17 [43] of the R-package. All heat maps depicting the expression levels of transcripts across the four tissue types were created using Heatmap2.0 of R-package.

The annotation of de novo transcriptome assemblies of *A. japonicum* was performed using a Blastx-based homology search against the NCBI-nr database with an E-value cutoff < 10^–5^, and the top Blastx hit was used to assign sequence description and putative functionality to the transcripts. Further, OmicsBox v3.1.2 was used to obtain GO terms, EC number, and KEGG pathway-based annotation for the transcripts of *A. japonicum*. For the identification of transcripts involved in the biosynthesis of diterpene alkaloid, the annotated transcriptome assembly was screened for the homologs of the associated enzymes, and candidate transcripts were selected that had a length greater than 500 bps and sequence similarity with top Blastx hit of over 70%.

### KEGG pathway enrichment analysis

KEGG pathway enrichment analysis was performed as described previously [11]. Briefly, all the assembled transcripts of *A. japonicum* were mapped to the KEGG pathway database and was selected as the reference set. Further, the highly expressed transcripts of the mother root and daughter root mapped on to the pathway were selected as the test set. Subsequently, hypergeometric test was used to calculate the *p* value and identify the enriched KEGG pathway using the following formula:

$$\text{P }\left(\text{X}=\text{x}\right)=h \left(x;n,M,N\right)=\frac{\left(\begin{array}{c}M\\ x\end{array}\right) \left(\begin{array}{c}N-M\\ n-x\end{array}\right)}{\left(\begin{array}{c}N\\ n\end{array}\right)}$$

Note: *x* represents the number of unigenes mapped to a certain KEGG pathway in the test set, *n* represents total number of unigenes in the test set mapped to KEGG pathway, *M* represents the number of unigenes in the reference set mapped to a certain KEGG pathway, and *N* represents the total number of unigenes in a reference set mapped to the KEGG pathway. Rich factor represents the ratio of the test gene number to the reference gene number for a specific pathway.

### Comparative transcriptome analysis of *Aconitum japonicum*, *Aconitum carmichaelii*, and *Aconitum heterophyllum* to infer phylogenetic relationship of three Aconitum species

The de novo transcriptome assemblies of *A. japonicum, A. carmichaelii,* and *A. heterophyllum* were translated into corresponding protein sequences by picking a translational frame that was used for annotation based on the Blastx results or resulting in longest amino acid sequence compared to rest translation frame. The translated proteins thus obtained were used to identify conserved orthogroups between the three plant species using OrthoFinder v2.5.5 [44], with its default parameters.

### Identification of simple sequence repeats (SSRs)

The de novo transcriptome assembly of *A. japonicum* was scanned for simple sequence repeats using the Microsatellite Identification Tool (MISA) with the search parameters described previously [11]. Briefly, we set the search parameters for maximum motif length group to recognize hexamers with each SSRs length-based category to have at least five repeats.

### Gene ontology (GO) enrichment analysis

Tissue-specific transcripts of *A. japonicum* with non-zero FPKM values as the test set were used against transcripts of *A*. *japonicum* with GO annotation as the reference set to perform GO enrichment analysis using OmicsBox tool v3.1.2 as described previously [11]. GO enrichment analysis based on Fisher’s exact test was performed with corrected *p* value cut-off set at 0.05. Furthermore, a hypergeometric test with Benjamini and Hochberg false discovery rate correction was also applied. GO enrichment analysis of transcripts of *A. japonicum* shared between *A. japonicum* and *A. carmichaelii* and absent in *A. heterophyllum* was performed using a similar approach to that described above.

### Integrative omics analysis of transcriptome and metabolome dataset of *Aconitum japonicum*

The average level of the peak intensity of all biological replicates of each MS/MS-validated metabolite and expression level of transcripts with FPKM value over five in at least one of the four tissues in the transcriptome data was used to perform Pearson correlation-based integration analysis of metabolome and transcriptome datasets of *A. japonicum*. The correlation relationships between metabolites and transcripts were visualized using correlation plot in R package.

### Phylogenetic analysis of candidate cytochrome P450s in *Aconitum japonicum*

The transcripts of *A. japonicum* denoted as cytochrome P450, having a sequence length of over 500 bps, and sequence similarity with the top Blastx hit of 70% was selected for phylogenetic analysis. These selected transcripts were translated to their corresponding protein sequences by selecting the translation frame that resulted in the longest amino acid sequence while starting with methionine using BLAST2GO. Protein sequences of cytochrome P450 annotated transcripts and previously characterized CYP450s from other plant species across different clans were aligned using the MUSCLE program v3.8.31 [45], and evolutionary distances were inferred using the maximum-likelihood method, based on a Jones–Taylor–Thornton (JTT) matrix-based model with bootstrap values obtained after 1000 replications using MEGA X software v10.2.6 [46].

## Results and discussion

### Tissue-specific accumulation of diterpene alkaloids in *Aconitum japonicum*

**Fig. 1:**
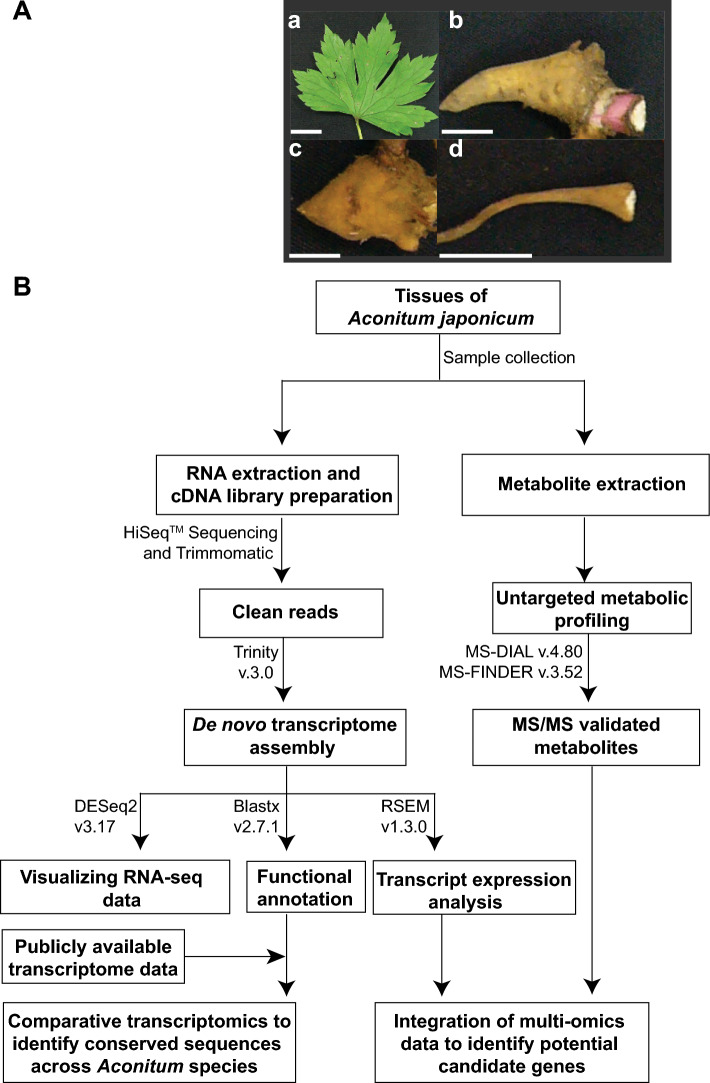
Overview of the experimental design and opted multi-omics analysis pipeline to establish high-quality omics resources for *Aconitum japonicum*. **(A)** Four tissues of *A. japonicum* were used for integrative omics study, including leaf **(a)**, mother root **(b)**, daughter root **(c)**, and rootlet **(d)**. The scale bar represents 1 cm. **(B)** Schematics of the opted multi-omics analysis pipeline used for the metabolome, transcriptome, and their integration analysis to identify potential candidate genes associated with diterpene alkaloids biosynthesis in *A. japonicum*

**Fig. 2:**
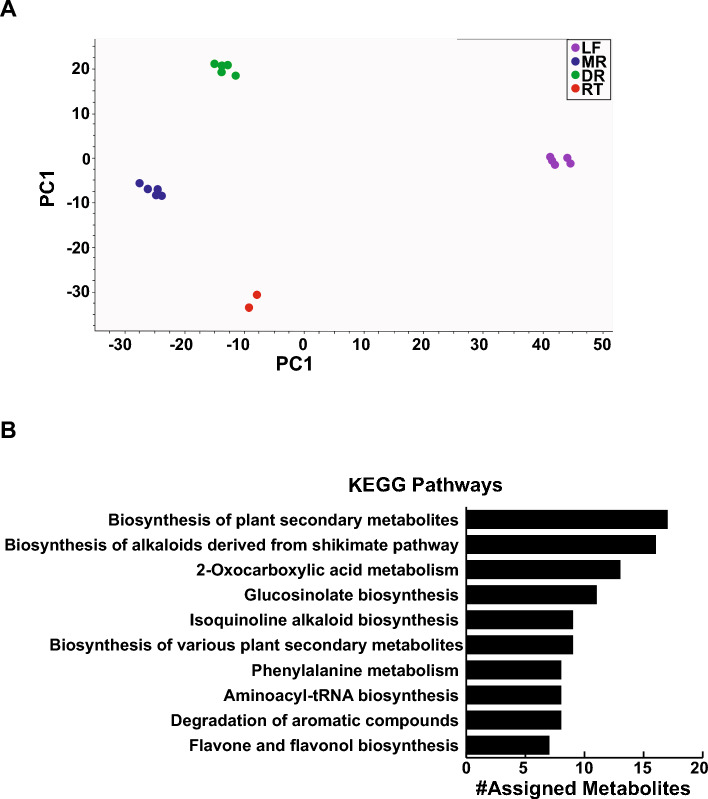
Metabolome resource of *Aconitum japonicum* established using untargeted metabolomic approach*. (A)* Unsupervised principal component analysis (PCA) using the metabolome datasets acquired for the four tissues of *A. japonicum.* **(B)** Top ten KEGG pathways based on the number of assigned metabolites. The mass features were mapped to the KEGG database, and the chemical identities were assigned based on the *m/z* similarity with a mass-error window of ± 10 ppm. Further, the KEGG pathways related to the assigned metabolites were extracted, the top ten of which are shown here. Abbreviations: LF (leaf), MR (mother root), DR (daughter root), RT (rootlet)

Specialized metabolites accumulate in specific tissues to fulfill distinct functions under various developmental and stress conditions [29, 47]. To capture diversity and distribution of phytochemical in *A. japonicum*, we performed untargeted metabolite profiling using a high-resolution UPLC–QTOF-MS/MS for four tissues, namely, leaf, mother root, daughter root, and rootlet **(**Fig. 1**, **Figure S1**)**. Metabolite profiling was performed in MS^1^ and data-dependent MS/MS mode, and the acquired raw data were pre-processed and used for peak detection and alignment using MS-DIAL as described previously [34, 35]. A total of 2,045 mass features were detected in the positive-ion mode, enabling multivariant data analysis and MS/MS-based structure prediction **(**Table S1**)**. Unsupervised principal component analysis (PCA) revealed tissue-wise classification for metabolite content of four tissues of *A. japonicum* along the PC1 and PC2 axes. Notably, leaf samples exhibited separation from the other tissues along the PC1-axis, while different root types were distinguished along the PC2-axis **(**Fig. 2A**)**. These findings suggest that the metabolomes of the selected tissues effectively encompass a diverse and distinct array of bioactive compounds across its tissues, thereby underpinning the therapeutic potential possessed by the different tissues of *A. japonicum.*

In an attempt to associate the metabolome datasets with comprehensive chemical databases and biochemical pathway information, we performed mapping using the KNApSAcK [48] and KEGG [49] databases for the acquired mass features. We assigned 533 and 123 mass features to KNApSAcK and KEGG databases, respectively **(Table S2)**. The top ten pathways, ranked by the number of mapped mass features, are shown in Fig. 2B. Notably, the top three KEGG pathways included “Biosynthesis of Plant Secondary Metabolites”, “Biosynthesis of Alkaloids Derived from Shikimate Pathway”, and “2-Oxocarboxylic Acid Metabolism”. The untargeted metabolome profiling and MS/MS-based metabolite annotation based on pathway mapping unveiled a plethora of metabolites, including flavonoids, phenylpropanoids, and anthocyanins, in addition to the known diterpene alkaloids within the metabolite space of *A. japonicum.* Comparison of our findings with established databases like KEGG and KNApSAcK revealed a significant overlap between the detected metabolites and known *Aconitum* secondary metabolites, thereby validating our analytical approach. Recently, a comprehensive untargeted metabolome profiling for over 150 plant species was published as RefMetaPlant, which included *A. carmichaelii* as one of the plant species [50]. The database reported 1,102 metabolites from *A. carmichaelii*. For *A. japonicum*, we identified over 2,000 mass features, with over 500 mass features being mapped to the KNApSAcK database. The challenges in terms of assigning chemical identities to a given mass feature is limited availability of MS/MS spectral datasets; nevertheless, the identified metabolites in this study were relative to what has been previously reported for other plant species. Comparing spectral profiles using the metabolome of *A. japonicum* will be useful to identify intermediates associated with aconitine biosynthesis. Additionally, tissue-wise accumulation trends offer key insights on the function of a metabolite toward plant physiology, which would be possible using the established metabolome resource of *A. japonicum* from this study. Moreover, the metabolite diversity reported in this study lay the groundwork for future investigations focused on discovery and structural validation of the bioactive metabolites and their corresponding pathways in *A. japonicum.*

**Fig. 3:**
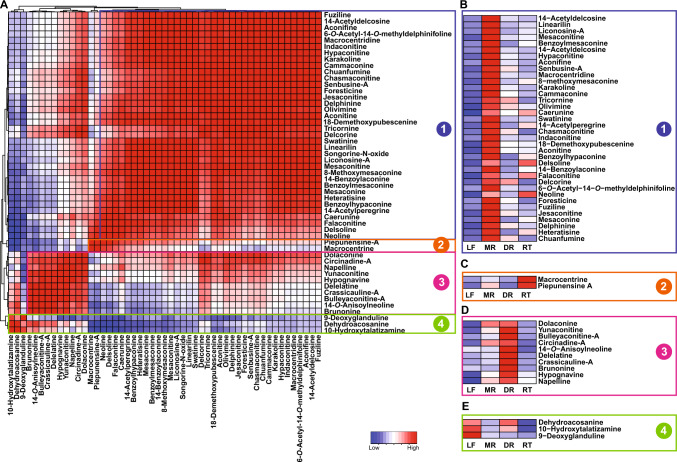
Correlation analysis and accumulation pattern for MS/MS fragmentation based annotated alkaloids in *Aconitum japonicum.* Pearson correlation coefficients were calculated between chemically assigned alkaloids using accumulation levels across four tissues of *A. japonicum,* and correlation scores are plotted as a heatmap with corresponding alkaloid names represented along the X- and Y-axes. Hierarchical clustering based on correlation scores for alkaloids formed four distinct groups, labeled as 1–4, and the accumulation levels are shown as metabolite clusters **(B–E)** across four tissues of *A. japonicum*. Abbreviations: LF (leaf), MR (mother root), DR (daughter root), RT (rootlet)

As a proof of concept to explore the established metabolome resource of *A. japonicum*, we used a combination of manual inspection and computational cheminformatics approach using MS-FINDER [51], and confirmed 160 metabolites using their MS/MS fragmentation spectra (Table S3). Among these annotated metabolites, we identified 51 diterpene alkaloids, with 40 belonging to the C_19_-type, known to be the predominant alkaloids of the toxic *Aconitum* species. The accumulation-based correlation analysis across four tissues of the MS/MS confirmed alkaloids revealed the presence of four distinct clusters **(**Fig. 3A**)**. Cluster 1, the biggest cluster with 36 diterpene alkaloids, exhibited high accumulation in the mother root and included the majority of the C_19_-type alkaloid, such as aconitine, mesaconitine, jesaconitine, and hypaconitine, among others **(**Fig. 3B**)**. The medicinal efficacy of *A. japonicum* can be directly linked to the presence of C_19_-type diterpene alkaloids. Therefore, the enhanced accumulation of these metabolites in the mother root, the tissue primarily used for medicinal purposes, reinforces the robustness of our metabolome data and the analysis pipeline. Cluster 2, comprising two alkaloids, namely macrocentrine and piepunensine A, were highly accumulated in rootlets, followed by the mother root **(**Fig. 3C**)**. Cluster 3 represented ten diterpene alkaloids, primarily of C_20_-type, including dolaconine, delelatine, and yunaconitine, among others, exhibited high accumulation in the daughter root **(**Fig. 3D**).** It is hypothesized that C_20_-type diterpene alkaloids are the precursor for the C_19_- and C_18_-type alkaloid found in *Aconitum* species [52]**.** Therefore, the high accumulation of C_20_-type diterpene alkaloids in the daughter root may signal its readiness for detachment, poised to serve as the mother root for the next propagation. Cluster 4, comprising three metabolites, namely dehydroacosanine, 10-hydroxytalatizamine, and 9-deoxyglanduline, exhibited the highest accumulation in the leaves of *A. japonicum* **(**Fig. 3E**).** While 10-hydroxytalatizamine and 9-deoxyglanduline have been previously isolated from the aerial parts of plants from the Ranunculaceae family [53, 54], dehydroacosanine has been reported to be present in the whole plant metabolic extract of *A. barbatum* [55]. Therefore, the high accumulation of metabolites from cluster 4 in the leaves, as identified in our study, aligns with prior reports that had hinted at their presence in the aerial part. This consistency in our results with other plants from the Ranunculaceae plants demonstrates the potential role of these phytochemicals in plant physiology, including their potential effects on predators.

Our metabolome resource data, thus established, effectively captured the overall metabolite diversity in *A. japonicum,* with diterpene alkaloids emerging as the key metabo-constituents. The results reaffirmed the abundance of C_19_-type diterpene alkaloids in toxic *Aconitum* species. Additionally, we observed high accumulation of diterpene alkaloids in both the mother and daughter roots, consistent with these tissues being primarily used for medicinal purposes [5]. The tissue-specific accumulation of key metabolites in *A. japonicum* suggests tissue-wise biosynthesis and a higher order and unexplored transport mechanisms for phytochemical as key player in shaping this metabo-phenotype. Therefore, tissue-wise expression analysis and gene-metabolite association analysis could reveal candidates involved in the biosynthesis of diterpene alkaloids in *A. japonicum.* Furthermore, results from this study, along with the comprehensive metabolome established herein, will undoubtedly help in further investigations and structural validation of metabolites of interest in *Aconitum* species.

### Homology-based functional characterization for de novo transcriptome assembly for *Aconitum japonicum*

In the absence of genomic resources of *A. japonicum*, we performed deep coverage RNA-seq-based transcriptome profiling using the same tissues as used for metabolome analysis. Total RNA was extracted from the four tissues, and the corresponding cDNA libraries were sequenced using Illumina HiSeq™ 2000 platform. Subsequently, the sequencing reads were pre-processed to remove adapter sequences, low-quality reads, and shorter reads (< 50 base pairs), resulting in 44,107,721 clean paired-end reads. The average Phred score, which is a measure of the quality of the sequence reads, for each library exceeded 36. Preprocessing RNA-seq datasets using Trimmomatic program [38] resulted in < 1% of the total sequence data being dropped, indicating the suitability of our RNA-sequencing for deriving de novo transcriptome assembly (Table S4). Subsequently, pre-processed RNA-seq reads, thus obtained from individual tissue, were pooled together to derive de novo transcriptome assembly using Trinity program v.3.0 [39], followed by application of CD-HIT-EST [40] to remove sequence redundancies. The resulting de novo transcriptome assembly of *A. japonicum* consists of 129,760 transcripts, with an average length and median length of 600 and 368 bps, respectively, and an N50 value of 811bps **(**Table 1**)**. The length of the assembled transcripts varied from 224 to 15,777 bps, with sequence lengths of 19,288 and 1,261 unigenes greater than 1000 and 3000 bps, respectively (Figure S2A). SSRs’ identification driven by transcriptome assemblies has been successfully used to validate polymorphisms across multiple genotypes in different plant species [56–58]. We used de novo transcriptome assembly of *A. japonicum* and identified 18,572 SSRs candidates, with 2,092 transcripts containing more than one SSRs (Figure S2B, Table S5). The results would be useful to develop EST-SSR markers of *A. japonicum* for determining genetic variations in establishing *A. japonicum* genotypes for medicinal purposes and its sustainable production.

**Table 1:** Transcriptome assembly statistics for *A. japonicum*

Description	Transcripts
Number of transcripts	129,760
Total assembled bases	77,948,525
Average length (bps)	600.71
Median length (bps)	368
Maximum length (bps)	15,777
Minimum length (bps)	224
N50 (bps)	811
GC content (%)	42.65

The de novo transcriptome assembly of *A. japonicum* were subjected to a Blastx [59] search against the NCBI non-redundant (nr) database (http://www.ncbi.nlm.nih.gov, 8 May 2020, date last accessed; formatted on February 2022) for sequence homology-based annotation and characterization. Blastx results showed the majority of the transcripts having significant homology with their corresponding matched sequences in the database (Figure S3A, Table S6). The top-hit obtained for each query was used for the transcripts’ annotation. In total, 59,691 transcripts were annotated, with 43,152 transcripts sharing above 70% sequence similarity with their corresponding Blastx search hit (Figure S3B, Table S6). Further, 54% of the transcripts of *A. japonicum* remained unannotated, which potentially include novel, uncharacterized genes, or isoforms lacking closely related sequences in the database (Figure S3C, Table S6). Species distribution plot based on Blastx-top-hit identified *Vitis vinifera*, *Populus trichocarpa*, and *Ricinus communis* as the top three species exhibiting high-sequence similarity with the assembled transcripts of *A. japonicum* (Figure S3D). *Coptis japonica*, a member of Ranunculaceae family as *A. japonicum*, was also among the top-hit species showing high-sequence similarity with the assembled transcripts*.* Annotation distribution results further highlight the limited genomic availability for plants producing similar metabolite classes as *Aconitum.*

**Fig. 4:**
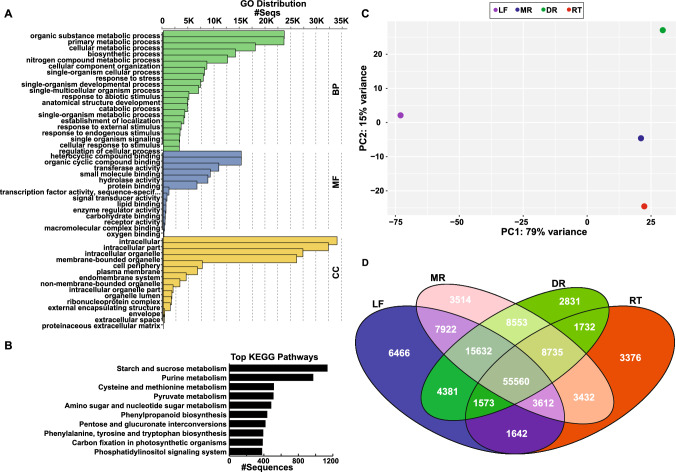
Transcriptome analysis across four tissues of *Aconitum japonicum* **(A)** Gene ontology (GO) annotation for *A. japonicum* transcriptome assembly into three major classifications, namely, BP (biological processes), MF (molecular functions), and CC (cellular components) based on Blast2GO-based analysis. **(B)** Top ten KEGG pathways based on the number of transcripts being assigned. **(C)** Unsupervised principal component analysis using transcriptome datasets generated for four tissues of *A. japonicum.* **(D)** Venn diagram representing the overall distribution of transcripts (FPKM > 0) across four tissues of *A. japonicum.* Abbreviations: LF (leaf), MR (mother root), DR (daughter root), RT (rootlet)

For a comprehensive characterization of the transcriptome assembly, annotations from Blastx search against NCBI-nr database were further analyzed using OmicsBox software v3.1.2 (https://www.biobam.com/) to assign functional descriptions, Enzyme Commission (EC) numbers, Gene Ontology (GO) terms, and KEGG pathways. GO terms were assigned to 48,756 transcripts across three broad categories: biological processes, metabolic processes, and cellular components. Within the biological process GO category, the top GO terms included organic substance metabolic process, primary metabolic process, cellular metabolic process, biosynthetic process, and nitrogen compound metabolic process (Fig. 4A, Table S6). The molecular function category comprised of heterocyclic compound binding, organic cyclic compound binding, transferase activity, small molecule binding, and hydrolase activity as the top five GO terms. In the cellular component category, intracellular, intracellular part, intracellular organelle, membrane-bound organelle, and cell periphery represented the most abundant GO term. To gain insights into the metabolic pathways being present in *A. japonicum*, the assembled transcripts were mapped to the KEGG database, resulting in 8,349 transcripts assigned to 154 KEGG pathways. The top ten pathways based on the number of assigned transcripts are shown in Fig. 4B. Among these pathways, 1136 transcripts were mapped to starch and sucrose metabolism, 970 transcripts to purine metabolism, and 515 transcripts were mapped to cysteine and methionine metabolism. Additionally, 271 transcripts were assigned to isoquinoline alkaloid biosynthesis, while 128 and 59 transcripts were mapped to terpenoid backbone biosynthesis and diterpenoid biosynthesis, respectively.

GO analysis categorized a significant proportion of the assembled transcripts across well-established functional groups, offering important insights into the biological processes, molecular functions, and cellular components being represented within the transcriptome of *A. japonicum.* Further, KEGG pathway mapping revealed extensive coverage of key metabolic pathways being expected, thus, highlighting the capability of de novo transcriptome assembly of *A. japonicum* in capturing its essential molecular mechanisms. These results demonstrate that the quality of the *A. japonicum* transcriptome is highly comparable to that of other non-model plant species [11, 32, 33, 37], thus providing a solid foundation to explore its genetic and metabolic landscapes.

### Expression analysis identifies potential candidate genes associated with diterpene alkaloid biosynthesis in *Aconitum japonicum*

Within a plant tissue, transcriptionally active genes encompass both shared transcripts across tissues responsible for general growth and development, and distinct sets of active transcripts exclusive to that tissue, which play a pivotal role in facilitating specialized tissue functions, including biosynthesis, regulation, and transportation of specialized metabolites [37]. To gain a comprehensive view of active biological processes and the associated transcripts within and across the four tissues of *A. japonicum*, we utilized the RSEM program [42] to determine transcript expression. Subsequently, we performed PCA using expression abundance dataset to elucidate overall relationship between four tissues of *A. japonicum* based on active transcripts expression levels. PCA plot showed a distinct separation of the four tissues into well-defined groups. While the leaf and different root types showed separation along the PC1-axis, accounting for 79% variance, different root types were separated along the PC2-axis with 15% variance **(**Fig. 4C**)**. Further, PCA for transcriptome datasets showed a similar clustering pattern as that of for metabolome datasets, suggesting a strong correlation between the transcript’s expression and metabolite accumulation. PCA analysis for four tissues of *A. japonicum*, therefore, clearly suggests the transcripts abundance dataset consists of tissue-specific signature transcripts, and the overlap of transcripts expression across tissues can be related to the tissue-type under investigation. Among the four tissues of *A. japonicum*, leaf, mother root, daughter root, and rootlet, we identified 96,788, 106,960, 98,997, and 79,662 transcriptionally active transcripts (FPKM > 0), respectively **(**Fig. 4D**)**. While 55,560 transcriptionally active transcripts were shared across the four tissues, 6,466, 3,514, 2,831, and 3,376 transcripts were tissue-specifically and expressed in the leaf, mother root, daughter root, and rootlet, respectively (Table S7). Moreover, a total of 64,295 active transcripts were shared between different root types. In particular, the mother root and daughter root, the two most important tissues of *A. japonicum* for its medicinal properties and commercial values, exhibited a significant overlap with 88,480 active transcripts shared between them*.* KEGG pathway enrichment analysis using highly expressed transcripts (FPKM > 100) in these two medicinally significant tissues revealed several of the KEGG pathways attributed to primary metabolism, including starch and sucrose metabolism, tryptophan metabolism, purine metabolism, and pyrimidine metabolism, being enriched (Figure S4). Interestingly, the terpenoid backbone biosynthesis, which synthesizes the precursor GGPP for the diterpene alkaloid biosynthesis, was also enriched in both the medicinally valuable tissues. 

**Fig. 5:**
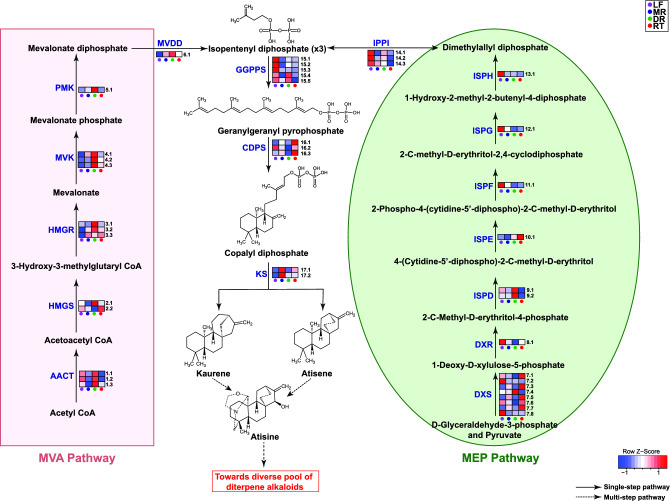
Schematics for the putative biosynthetic pathway of diterpene alkaloids in *Aconitum japonicum* and the tissue-wise expression for the assigned candidate genes to the biosynthesis pathways. Abbreviations: MVA pathway (mevalonate pathway), MEP pathway (methylerythritol pathway), LF (leaf), MR (mother root), DR (daughter root), RT (rootlet), AACT (acetoacetyl-CoA thiolase), HMGR (3-hydroxy-3-methylglutaryl-CoA reductase), HMGS (3-hydroxy-3-methylglutaryl-CoA synthase), MVK (mevalonate kinase), PMK (phosphomevalonate kinase), MVDD (mevalonate diphosphate decarboxylase), DXS (1-deoxy-d-xylulose 5-phosphate synthase), DXR (1-deoxy-d-xylulose 5-phosphate reductoisomerase), ISPD (2-C-methyl-d-erythritol 4-phosphate cytidylyltransferase), ISPE (4-(cytidine-5′-diphospho)-2-C-methyl-d-erythritol kinase), ISPF (2-C-methyl-d-erythritol 2,4-cyclodiphosphate synthase), ISPG ((E)-4-hydroxy-3-methylbut-2-enyl diphosphate synthase), ISPH ((E)-4-hydroxy-3-methylbut-2-enyl diphosphate reductase), IPPI (isopentenyl diphosphate isomerase), GGPPS (geranylgeranyl pyrophosphate synthase), CDPS (*ent*-copalyl diphosphate synthase), and KS (kaurene synthase)

Diterpene alkaloids, majorly consisting of the C_19_-type, are pivotal constituents of the *A. japonicum* metabolome, attributing to its medicinal properties [1]. As such, diterpene alkaloids remains at the primary focus for enhancing value addition and species improvement for medicinal purposes in *Aconitum* species. While the biosynthetic pathway of diterpene alkaloids remains largely unknown, the precursor molecules, GGPP, are derived from isopentenyl diphosphate (IPP) biosynthesized via both mevalonate (MVA) and methylerythritol (MEP) pathway [11, 15]. We used a combination of homology-based annotation, KEGG pathway and EC classifications, and strict criterion (length > 500 bps and similarity > 70%) to annotate 13 and 18 transcripts predicted to correspond to the known enzymes from the MVA and MEP pathway, respectively. Subsequently, we explored qualitative expression of transcripts annotated as enzymes associated with MVA or MEP pathway across four tissues. Most transcripts encoding enzymes from the MVA pathway were highly expressed in the daughter root **(**Fig. 5**)**, while homologs of enzymes 3-hydroxy-3-methylglutaryl coenzyme A reductase (HMGR), mevalonate kinase (MVK), and diphosphomevalonate decarboxylase (MVDD) showed relatively higher expression in the mother root. HMGR has long been considered as a rate-limiting enzyme for the MVA pathway, and its association with terpenoid biosynthesis has been widely reported [12, 60], which may suggest its critical role in the biosynthesis of diterpene alkaloids in *A. japonicum*. As expected for the plastidial MEP pathway, while all enzymes’ homologs were highly expressed in the leaf, the expression level of homologs associated with bottleneck enzyme, 1-deoxy-D-xylulose-5-phosphate synthase (DXS), and isopentenyl diphosphate isomerase (IPPI) showed relatively higher expression in the mother root and daughter root [12], suggesting subtle regulatory patterns in this pathway.

In the initial stage of diterpene alkaloid biosynthesis, three molecules of IPP undergo condensation reaction catalyzed by the enzyme geranylgeranyl pyrophosphate synthase (GGPPS), to form GGPP. GGPP then undergoes bicyclization in the presence of terpene synthases- copalyl diphosphate synthase (CDPS), and kaurene synthase or kaurene synthase like (KS/KSL) enzymes to form kaurene or atisene. Further, kaurene or atisene molecules are oxidized and hydroxylated in the subsequent steps as parallel reactions and form the atisine skeleton via incorporation of β-ethanolamine moiety [26]. Kumar et al. [61] previously reported that kaurene undergoes oxidation and hydroxylation in the presence of enzyme kaurene oxidase (KO) and kaurene hydroxylase (KH), respectively, to form steviol, which through incorporation of β-ethanolamine moiety leads to the formation of atisine. While we identified 19 transcripts predicted to be annotated as known enzymes from diterpene alkaloid biosynthesis pathway, namely GGPPS, CDPS, KS, and KO, homologous transcripts corresponding to kaurene hydroxylase (KH), a key enzyme implicated in the hydroxylation of kaurene to produce steviol, were conspicuously absent in our transcriptome dataset of *A. japonicum*. Intriguingly, the artificial sweetener steviol, resulting from this enzymatic process, was not only absent in our metabolome dataset but has also not been previously reported in the toxic *Aconitum* species. The expression levels for both the homologs corresponding to KS, class I diterpene synthase, were highly expressed in the mother root together with KO (Fig. 5, Table S8). The significantly high expression level of KS and KO, together with the absence of KH, may suggest that while the oxidation reaction toward biosynthesis of atisenol from atisene may be catalyzed by KO, the hydroxylation reaction is catalyzed by an enzyme other than KH, which remains unknown.

### Comparative transcriptome analysis to explore orthologous relationships among three *Aconitum* species

Comparative transcriptome analyses of closely related species have been employed to identify conserved transcripts involved in the biosynthesis of specialized metabolites in various plant species with distinct metabolic profiles, including *Panax* [37], and *Ilex* [62], among others. While plants from *Aconitum* genus are generally recognized for their high toxicity, *Aconitum heterophyllum,* primarily found in the Himalayan region, are significantly less toxic. This can be attributed to the predominance of C_20-_type diterpene alkaloids in *A. heterophyllum* in contrast to toxic *Aconitum* species such as *A. japonicum* and *A. carmichaelii* accumulating C_19_-type diterpene alkaloids as their signature metabolites [1, 61]. We used OrthoFinder for comparative transcriptome analyses using de novo transcriptome assembly for *A. japonicum* (this study), previously published transcriptome assembly for *A. carmichaelii*, and de novo transcriptome assembly (re-established in this study) using RNA-seq data from a previously published study for two tissues of *A. heterophyllum* were used for this analysis [11, 15].

OrthoFinder based comparative transcriptome analysis identified 38,828, 38,339, and 24,433 orthogroups in *A. japonicum, A. carmichaelii, and A. heterophyllum*, respectively, with 17,787 orthogroups containing at least one transcript from all three species (Figure S5A, Table S9). Within the subset of toxic *Aconitum* species transcriptome, 17,467 orthogroups represented at least one transcript from *A. japonicum* and *A. carmichaelii* while none from *A. heterophyllum*. In total, we identified 20,943 transcripts specific to the toxic species (Table S9), which include transcripts vital for the biosynthesis and regulation of specialized metabolites that are associated with the characteristic toxic biochemicals. GO term enrichment analysis identified defense response as one of the significant GO terms being enriched (Figure S5B). When comparing the transcripts of *A. japonicum*, annotated as enzymes involved in the diterpene alkaloid biosynthesis, with those of *A. carmichaelii*, we observed that nearly all transcripts identified from the *A. japonicum* transcriptome dataset had significant matches in the *A. carmichaelii* dataset. Among the 41 identified transcripts of *A. japonicum* predicted to encode enzymes involved in the diterpene alkaloid biosynthesis pathway leading to the kaurene/atisene core structure (Fig. 5, Table S8), 34 were included in the orthogroups shared with *A. carmichaelii* (Table S10). Moreover, the trend of the expression for most of the shared transcripts between root and leaf tissues was similar between these two plant species.

The comparative transcriptome analysis, presented here, provides a comprehensive perspective on the conserved sequences across two toxic *Aconitum* species, and contributes valuable insights into the shared genetic underpinnings of diterpene alkaloid biosynthesis. These transcripts could serve as potential candidates for further functional characterization and to explore their contribution to the biosynthesis of specialized metabolites. While the comparative transcriptome analyses yielded significant insights into the orthogroups’ distributions among *Aconitum* species, it is important to note that the use of de novo transcriptome assemblies presents certain limitations. These assemblies are inherently uneven and may not represent the entirety of the genome, leaving potential room for partially reconstructed transcripts. Further, the absence of transcripts in *A. heterophyllum* could well be due to fewer tissues used for constructing its de novo transcriptome assembly, which needs to be taken into consideration while selecting genes for further analysis. Analyzing these results with integrative multi-omics data is essential to identify strong candidate genes with a role in the biosynthesis of diterpene alkaloids. Consequently, further validation through complementary genomic or proteomic approaches is necessary to corroborate these findings.

### Data-driven integration of metabolome and transcriptome to decipher the biosynthesis of diterpene alkaloids in *Aconitum japonicum*

Only the initial steps of the diterpene alkaloids biosynthesis have been known in *Aconitum* species. Therefore, the utilization of the advanced biotechnological toolkits, which relies on the complete knowledge of the biosynthetic pathway, for enhancing the production of bioactive metabolites is severely limited. Several studies in the past have highlighted the importance of co-expression-based integration analysis of metabolome and transcriptome datasets as a valuable tool to identify the functionally active genes across different plant species [63]. Therefore, in this study, we used a gene-metabolite integration approach to get insights into the known and unknown molecular components involved in the biosynthesis of diterpene alkaloids in *A. japonicum.* Pearson-based correlation analysis was performed for MS/MS-validated metabolites (Table S3) and annotated transcripts that were highly expressed (FPKM > 5) in at least one of the four tissue of *A. japonicum* (Table S11). Results of integrative omics analysis identified metabolite–transcripts relationships in the form of two distinct large clusters, cluster 1 and cluster 2, respectively (Figure S6A, Table S11). While cluster 1 was dominated by metabolites annotated as diterpene alkaloids, cluster 2 majorly contained metabolites annotated as phenylpropanoids and anthocyanins. Since the primary objective of this study was to identify candidate molecular components associated with the biosynthetic pathway of diterpene alkaloid in *A. japonicum,* we next conducted an in-depth investigation of the diterpene alkaloid cluster (cluster 1) to understand the type of enzymes being represented by the transcripts included in the cluster 1. Cluster 1 included 2,517 transcripts together with 47 metabolites (Table S11). The expression level of all the transcripts included in cluster 1 was highest in mother root (Figure S6B). Similarly, the accumulation levels of diterpene alkaloids present in cluster 1 were highly accumulated in the mother root, followed by the daughter root of *A. japonicum* (Figure S6C). Interestingly, transcripts encoding several of the important enzyme classes, such as CYPs, KS, KO, transcription factors, acyltransferase, and methyltransferase, were co-expressed with these metabolites and may suggest critical enzymes participating in the biosynthesis and diversification of these diterpene alkaloids. Further utilizing phylogenetic analysis of the identified candidate genes of *A. japonicum* with functionally characterized candidate genes will provide valuable insights into their respective functions.

### Phylogenetic analysis of transcripts annotated as cytochrome P450 and co-clustered in the diterpene alkaloid cluster

Terpenoids represent the most abundant group of specialized plant metabolites, with tens of thousands of known structures spread across the entire plant kingdom [64]. The structural diversity of terpenoids relies on the modification of specific chemical groups, rearrangements of the scaffold skeletal structure, and post-modification reactions [65]. In general terms, the biosynthesis of terpenoids first involves a terpene synthase that acts on a diphosphate substrate, followed by a cytochrome P450 that participates in the functionalization of the core terpenoid moiety, thereby contributing to its known diversity [66]. To identify potential cytochrome P450 candidate genes involved in the biosynthesis of diterpene alkaloids in *A. japonicum,* we focused on cytochrome P450s that were highly correlated with the diterpene alkaloid cluster (Table S11). Among the highly correlated CYPs, 22 exhibited length and percentage similarity with its corresponding Blast hits over 500 bps and 70%, respectively. Furthermore, five of these CYPs were annotated as enzymes associated with different primary metabolic processes, while 17 were putatively annotated to participate in the secondary metabolic processes (Table S11). Intriguingly, all the 17 identified CYPs showed the highest expression in the mother root followed by daughter root, with 16 of them included in the orthogroups as identified through OrthoFinder analysis (Table S12). Subsequently, we performed phylogenetic analysis of these 17 CYPs of *A. japonicum* together with selected functionally characterized CYPs from other plant species.

**Fig. 6:**
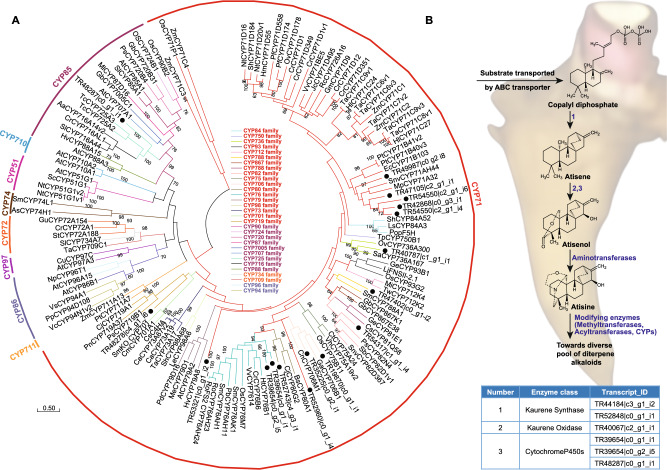
Putative novel genes associated with the biosynthesis of diterpene alkaloids and diversification in *Aconitum japonicum*. **(A)** Phylogenetic analysis of transcripts included in the highly correlated diterpene alkaloid cluster and annotated CYP450s. The cytochrome P450s having length and similarity over 500 bps and 70%, respectively, were used together with functionally characterized CYPs of the plant kingdom across different clans. The nucleotide sequences for unigenes annotated as CYP450s were translated and the corresponding protein sequences were aligned with functionally characterized CYPs using the MUSCLE program. The evolutionary history was inferred using the Maximum-Likelihood method and JTT matrix-based model with bootstrap values obtained after applying 1000 replications using the MEGA X program. Bootstrap values above 60% are shown here. Initial tree(s) for the heuristic search were obtained automatically by applying Neighbor-Join and BioNJ algorithms to a matrix of pairwise distances estimated using the JTT model, and then selecting the topology with superior log likelihood value. The tree is drawn to scale, with branch lengths measured in the number of substitutions per site. The scale bar represents 0.5 estimated nucleotide changes per sequence position. The accession number of all the functionally characterized CYPs are included as Supplementary Table. **(B)** Proposed biosynthetic pathway of diterpene alkaloids biosynthesis and diversification as revealed by the integration of the metabolome and transcriptome datasets of *A. japonicum*

Of the 17 CYPs discovered in *A. japonicum*, 16 were classified into various families within the CYP71 clan. The remaining one CYP, TR48287|c0_g1_i1, grouped with the CYP85 clan, alongside taxane hydroxylase, TcCYP725A2 and TcCYP725A3. **(**Fig. 6A**)**. Taxane hydroxylases catalyzes the hydroxylation reaction in the biosynthesis of diterpenoid, taxol, in the *Taxus* species [67]. Therefore, the grouping of TR48287|c0_g1_i1 within CYP85 clan suggests its potential involvement in the biosynthesis of diterpene alkaloids in *A. japonicum*. Two of the 16 CYPs, TR53321|c0_g1_i2 and TR52960|c0_g1_i4, formed an independent cluster within the CYP71 clan, while TR47402|c0_g1_i2 grouped with the CYP788 family of the CYP71 clan known for its involvement in the lignin biosynthesis [68]. Additionally, two CYPs, TR19870|c0_g1_i1 and TR35225|c0_g2_i1, and TR40787|c1_g1_i1 were clustered together with CYP706 family and CYP736 family, respectively, which are known to catalyze reactions leading to the biosynthesis of sesquiterpenes [68]. Another transcript of *A. japonicum*, TR54317|c1_g1_i4, was grouped with the CYP82 family of CYP71 clan, recognized for its role in the flavonoid biosynthesis across different plant species [68]. Two of the CYPs, TR48278|c4_g1_i6 and TR52743|c4_g3_i1, from *A. japonicum* clustered together with the CYP80 and CYP719 families, respectively. While cytochrome P450s included in the CYP80 family are known to catalyze C–C and C-O-phenol coupling reactions, the CYP719 family is known to catalyze the formation of methylenedioxy bridges toward the biosynthesis of BIAs [68]. Several of the lappaconitine and ranaconitine types of diterpene alkaloids contain methylenedioxy bridge. Therefore, the clustering of CYPs from *A. japonicum* with the functionally characterized CYPs from CYP719 family suggests a potential role for these CYPs in the diversification of *Aconitum* alkaloids derived from the atisine skeleton. TR49987|c0_g2_i8 was clustered together with the CYP71 family of the CYP71 clan known to participate in the biosynthesis of MIAs. Two of the CYPs, TR39654|c0_g1_i1 and TR39654|c0_g2_i5, were grouped with the CYP76 family. The CYP76 family has long been known to participate in the hydroxylation reaction toward several species’ biosynthesis of diterpene alkaloids [69, 70]. Moreover, CYP76M7 and CYP76M8 in rice are members of a gene cluster containing terpene synthases and participating in phytoalexin biosynthesis [71]. Further, TR39654|c0_g1_i1 was part of a single-copy orthogroups shared between *A. japonicum* and *A. carmichaelii,* and was absent in *A. heterophyllum* (Table S12)*.* Therefore, TR39654|c0_g1_i1 from *A. japonicum* represents the most promising candidate transcript catalyzing the initial hydroxylation reaction of atisene to form atisenol. Based on our integration of metabolome and transcriptome datasets followed by the phylogenetic analysis of candidate CYPs, we hereby propose the putative pathway for the biosynthesis of the atisine skeleton in *A. japonicum* **(**Fig. 6B**)**. The role of the promising candidate transcripts proposed in this study will need further functional characterization to validate their role in the biosynthesis of diterpene alkaloids.

## Conclusion

In this study, we established high-quality metabolome and transcriptome resources for *Aconitum japonicum*, which holds a unique position within the diverse *Aconitum* genus for its extensive pharmacological relevance in various traditional medicinal practices. The multi-omics approach focused on metabolomics and transcriptomics integration, has provided valuable insights into the genetic and molecular underpinnings of this medicinal plant. By focusing on four tissues, namely leaf, mother root, daughter root, and rootlet, we aimed to gain insight into the biosynthesis of aconitine-type diterpene alkaloids in *A. japonicum*. Through this study, we not only investigated the distinctive molecular signatures within each tissue, but also the integration of transcriptome and metabolome data allowed us to narrow down the candidate genes, which may act as the molecular architects responsible for the intricate pathways leading to the biosynthesis of therapeutic compounds in *A. japonicum*. Comparative transcriptome analysis with other *Aconitum* species highlighted the conserved molecular components among toxin producing species compared with the non-toxic *Aconitum* species. Our findings also lay the groundwork for future endeavors in harnessing the therapeutic potential of *A. japonicum*. The candidate genes identified in this study offer a key to unlocking the secrets of medicinal compound synthesis, guiding us toward sustainable and optimized approaches for medicinal plant cultivation. Furthermore, the comprehensive metabolome dataset, combined with the transcriptome data, offers an indispensable resource for understanding gene–metabolite relationships, providing a valuable platform for both functional genomics and metabolic engineering studies in *Aconitum* and related species.

## Supplementary Information

Below is the link to the electronic supplementary material.

Supplementary file1 (XLSX 383 KB)

Supplementary file2 (XLSX 123 KB)

Supplementary file3 (XLSX 61 KB)

Supplementary file4 (DOCX 61 KB)

Supplementary file5 (XLSX 26 KB)

Supplementary file6 (XLSX 10450 KB)

Supplementary file7 (XLSX 4270 KB)

Supplementary file8 (XLSX 17 KB)

Supplementary file9 (XLSX 3574 KB)

Supplementary file10 (XLSX 14 KB)

Supplementary file11 (XLSX 74841 KB)

Supplementary file12 (XLSX 32 KB)

Supplementary Figure 1: Root architecture of *Aconitum japonicum*. (A) Overview of the plant, showing the full root system. (B) Zoomed-in view of the root, highlighting the root architecture (PDF 1103 KB)

Supplementary Figure 2: Characterization of the de novo transcriptome assembly of *Aconitum japonicum*. (A) Length distribution of the assembled transcripts of *A. japonicum*. (B) Distribution of different repeat type classes of SSRs in the *A. japonicum* transcriptome assembly (PDF 831 KB)

Supplementary Figure 3: Blastx-based functional classification of the transcriptome assembly of *Aconitum japonicum*. (A) E-value distribution plot based on the Blast hits for the A. japonicum assembled transcriptome. (B) Sequence-similarity score distribution plot using top Blast hits used for the annotation of the de novo transcriptome assembly. (C) Bar chart representing data distribution of blast search-based annotation and Gene ontology assignment for *A. japonicum* transcriptome assembly using Blast2GO analysis. (D) Species distribution plot based on the top Blast hits for the assembled transcripts (PDF 506 KB)

Supplementary Figure 4: KEGG pathway enrichment of transcripts highly expressed (FPKM> 100) in (A) mother root, and (B) daughter root of *Aconitum japonicum* (PDF 399 KB)

Supplementary Figure 5: Comparative transcriptome analysis of *Aconitum japonicum* with two other *Aconitum* species, including *Aconitum carmichaelii* and *Aconitum heterophyllum*. (A) Venn diagram representing the distribution of orthogroups in *A. japonicum*, *A. carmichaelii*, and *A. heterophyllum* (B) GO enrichment analysis for transcripts specific to *Aconitum japonicum* and *Aconitum carmichaelii*. Using transcripts of *A. japonicum* having orthologous genes present only in *A. carmichaelii* as a test set and all the transcripts of *A. japonicum* with GO annotation as a reference set, gene ontology enrichment analysis was performed using Fisher’s exact test with the p value cut-off set as < 0.05 (PDF 634 KB)

Supplementary Figure 6: Correlation based integration analysis of the metabolome and transcriptome datasets of *Aconitum japonicum*. (A) Correlation coefficients were calculated between highly expressed annotated transcripts (FPKM>5) and the MS/MS-validated metabolites, and correlation scores are plotted as a heatmap with metabolites and transcripts represented along the X- and Y-axes, respectively. (B) The expression value of the transcripts included in the highly correlated cluster. The transcript name and expression data are included as the Supplementary Table 11. (C) The accumulation of the diterpene alkaloids included in the highly correlated cluster. Abbreviations: LF (leaf), MR (mother root), DR (daughter root), RT (rootlet) (PDF 3446 KB)

## Data Availability

The Illumina raw sequence reads, the de novo transcriptome assembly, annotations, and expression value for the unigenes have been deposited in NCBI’s Gene Expression Omnibus (GEO) and are available at GEO series accession number GSE275867.
