# Supplementary material for: Multi-omics analysis reveals tissue-specific biosynthesis and accumulation of diterpene alkaloids in Aconitum japonicum
Source: J Nat Med. 2025 Mar 20;79(3):499–516. doi: 10.1007/s11418-025-01881-y (PMC12058934; doi:10.1007/s11418-025-01881-y)
Supplement: Supplementary file 4 — Supplementary file4 (DOCX 61 KB) [file 11418_2025_1881_MOESM4_ESM.docx]

|  | **Leaf** | **Rootlet** | **Mother Root** | **Daughter Root** |
| --- | --- | --- | --- | --- |
| **Input Read Pairs** | 6,188,177 | 5,942,926 | 15,389,544 | 16,586,174 |
| **Both Surviving** | 5,854,184 (94.60%) | 5,608,520  (94.37%) | 14,452,436  (93.9%) | 15,571,379  (93.87%) |
| **Forward Only Surviving** | 314,581  (5.09%) | 315,325  (5.31%) | 888,352  (5.78%) | 961,500  (5.81%) |
| **Reverse Only Surviving** | 9,898  (0.16%) | 10,166  (0.17%) | 24,140  (0.15%) | 27,514  (0.16%) |
| **Dropped** | 9,244  (0.15%) | 8,915  (0.15%) | 24,616  (0.16%) | 25,781  (0.16%) |

**Supplementary Table 4- Summary of Trimmomatic output for *Aconitum japonicum***
