# Supplementary figures and images for: Multi-omics analysis reveals tissue-specific biosynthesis and accumulation of diterpene alkaloids in Aconitum japonicum
Source: J Nat Med. 2025 Mar 20;79(3):499–516. doi: 10.1007/s11418-025-01881-y (PMC12058934; doi:10.1007/s11418-025-01881-y)

**Supplementary Figure 1**

**A**

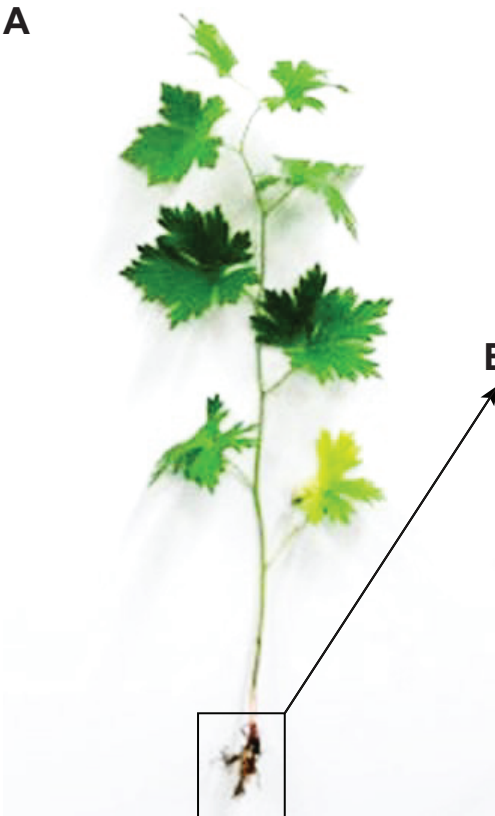

**B**

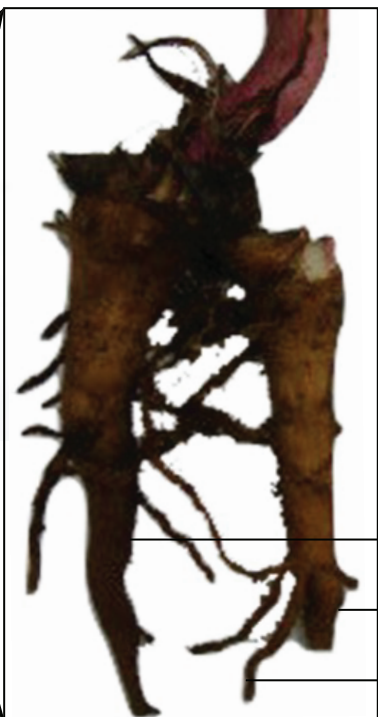

Mother Root (MR)

Daughter Root (DR)

Rootlet (RT)

Supplement: Supplementary file 13 — Supplementary Figure 1: Root architecture of Aconitum japonicum. (A) Overview of the plant, showing the full root system. (B) Zoomed-in view of the root, highlighting the root architecture (PDF 1103 KB) [file 11418_2025_1881_MOESM13_ESM.pdf]

Supplementary Figure 2

A

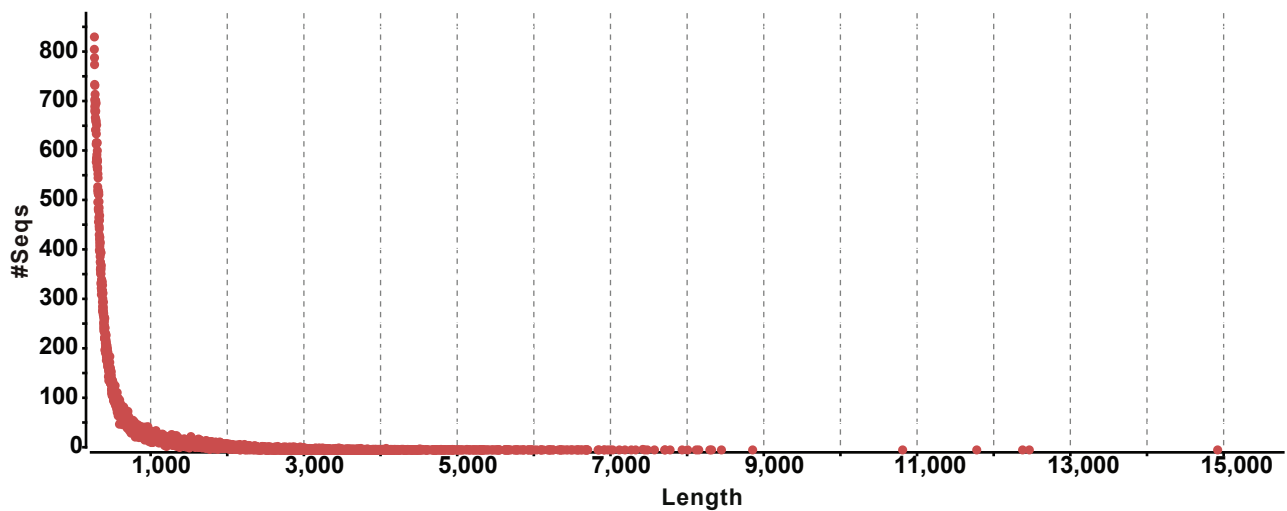

B

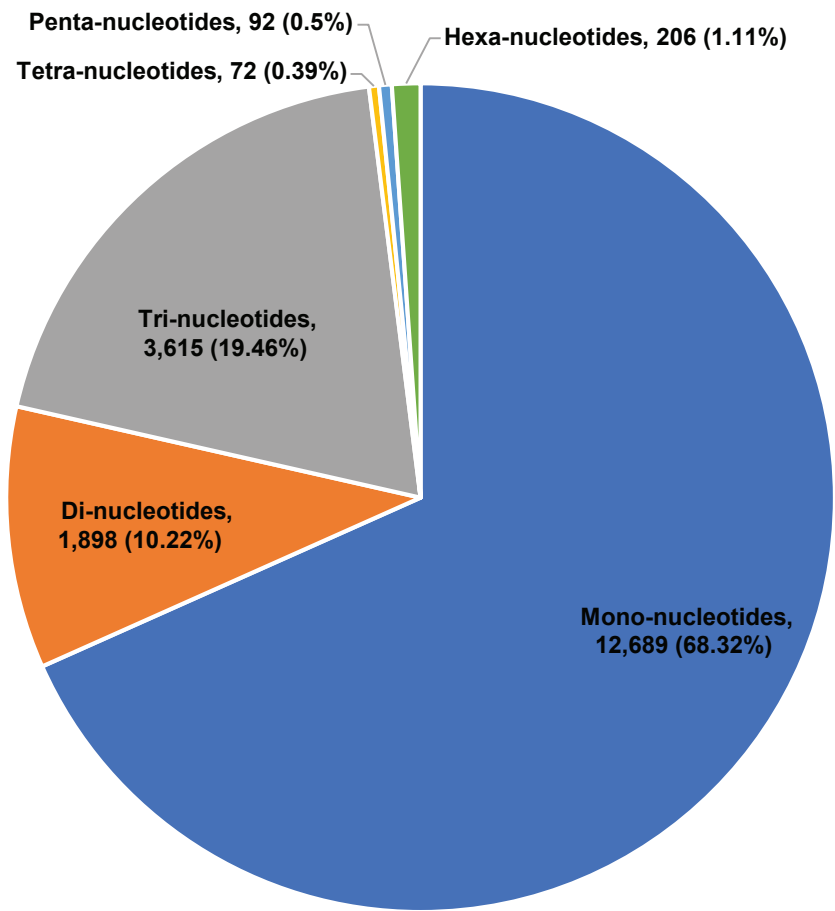

Supplement: Supplementary file 14 — Supplementary Figure 2: Characterization of the de novo transcriptome assembly of Aconitum japonicum. (A) Length distribution of the assembled transcripts of A. japonicum. (B) Distribution of different repeat type classes of SSRs in the A. japonicum transcriptome assembly (PDF 831 KB) [file 11418_2025_1881_MOESM14_ESM.pdf]

A

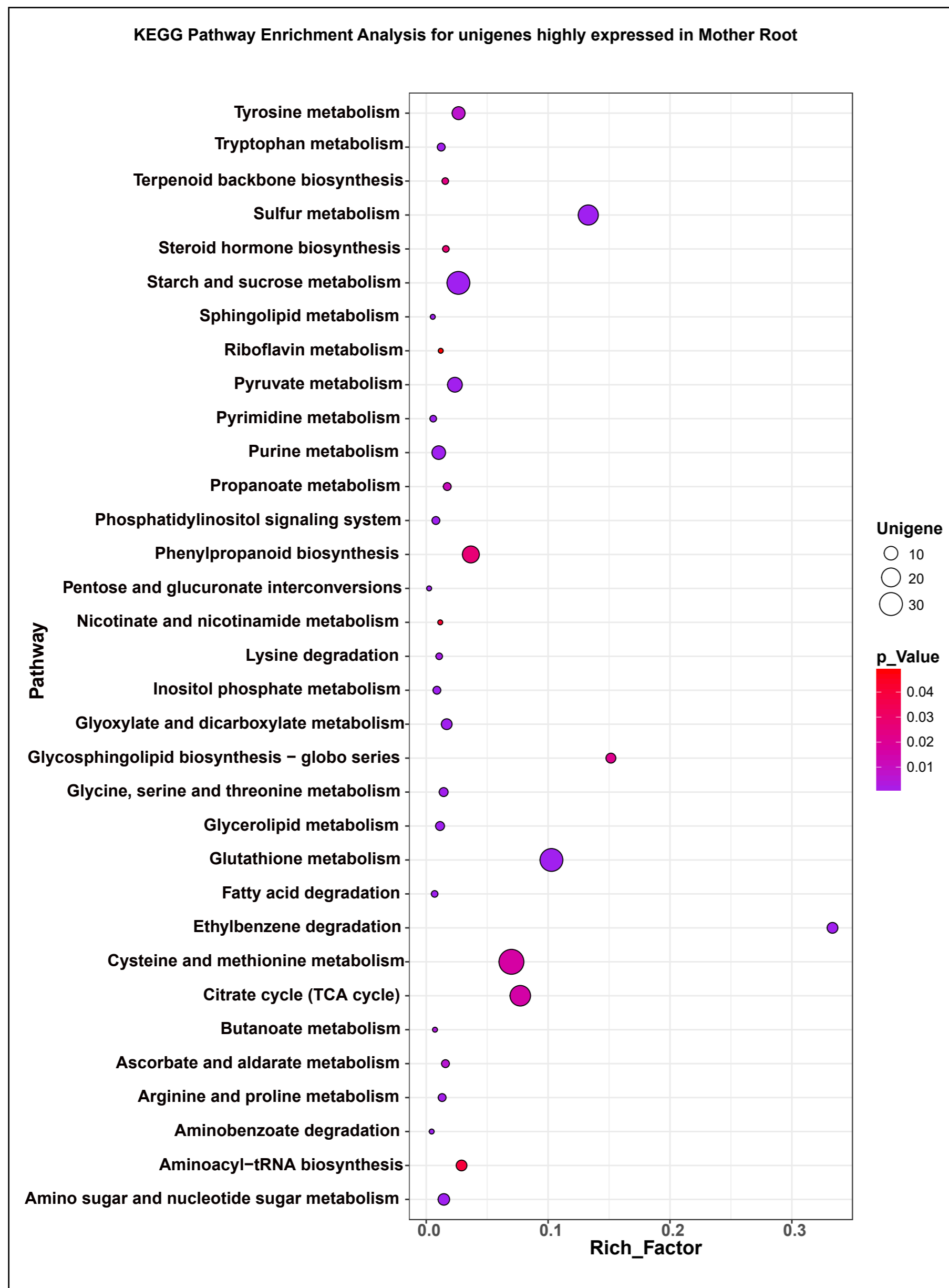

B

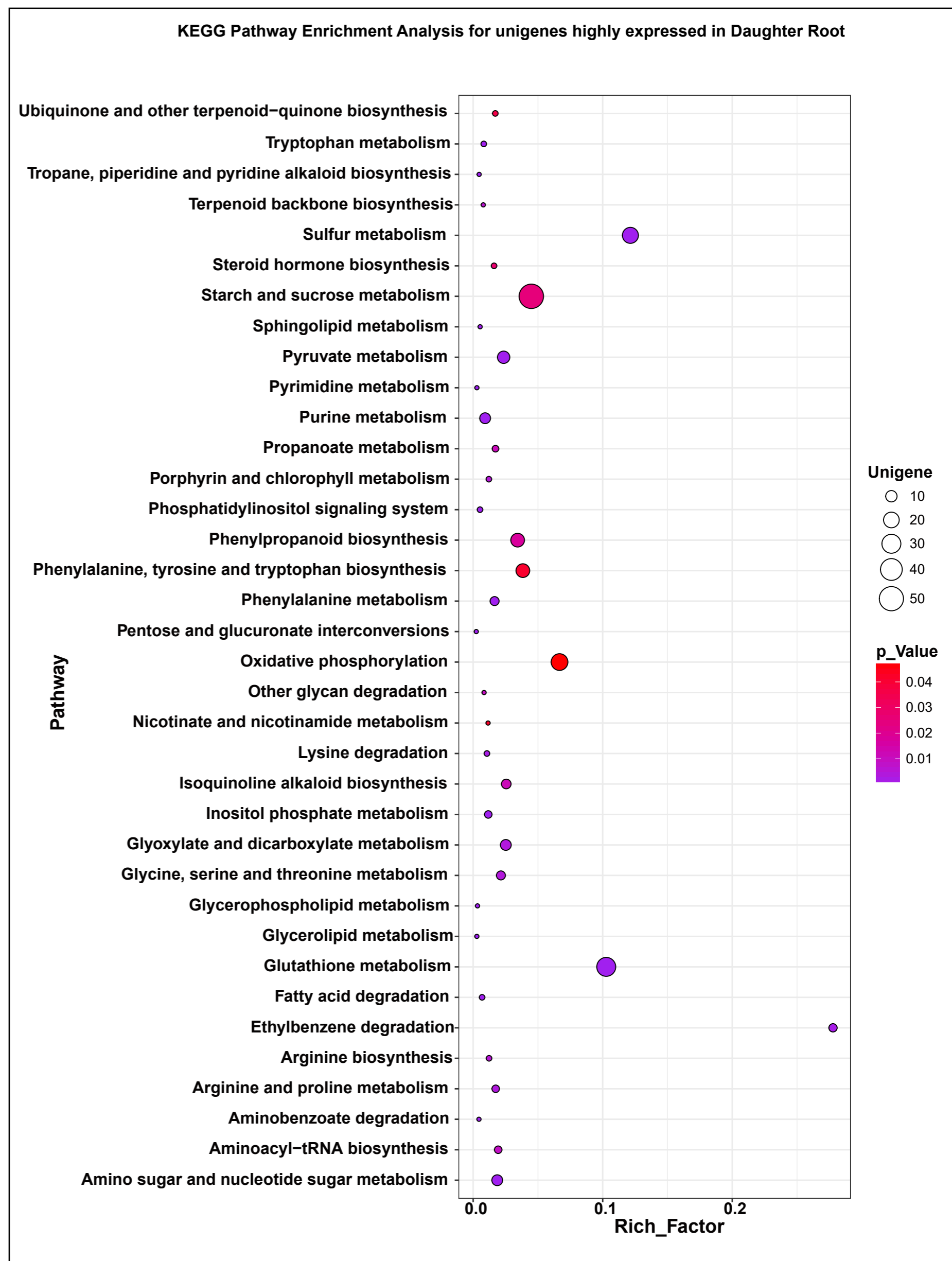

Supplement: Supplementary file 16 — Supplementary Figure 4: KEGG pathway enrichment of transcripts highly expressed (FPKM> 100) in (A) mother root, and (B) daughter root of Aconitum japonicum (PDF 399 KB) [file 11418_2025_1881_MOESM16_ESM.pdf]

Supplementary Figure 5

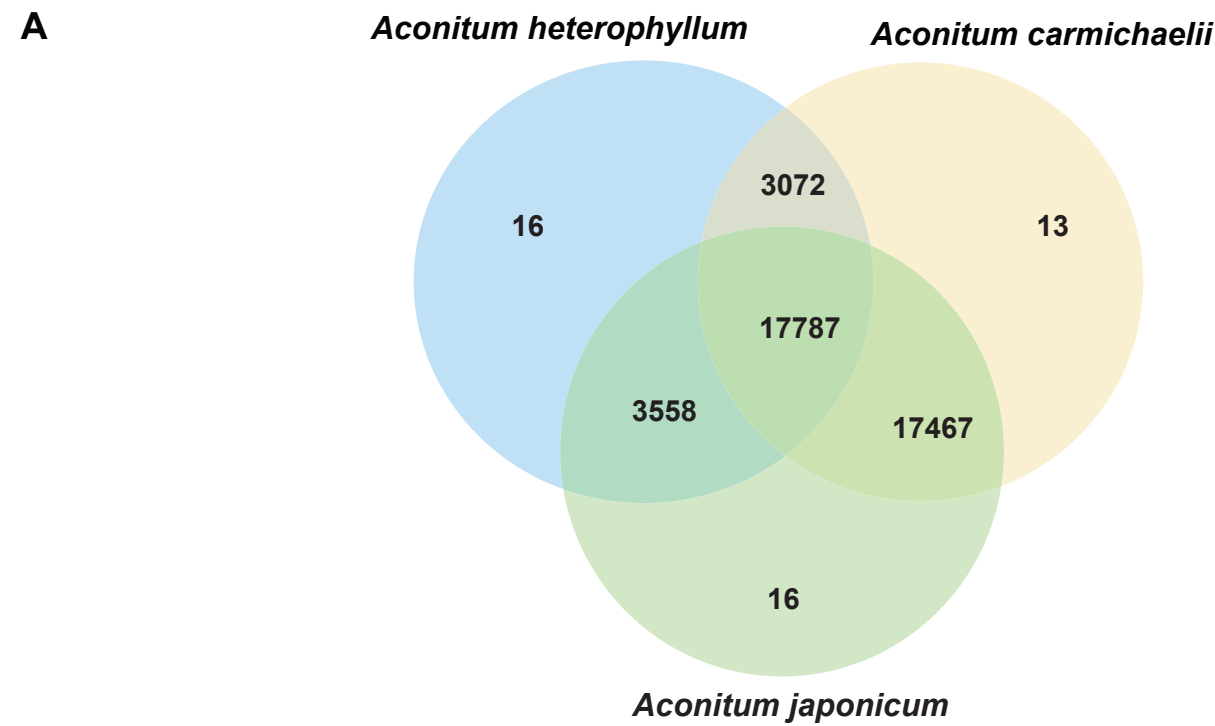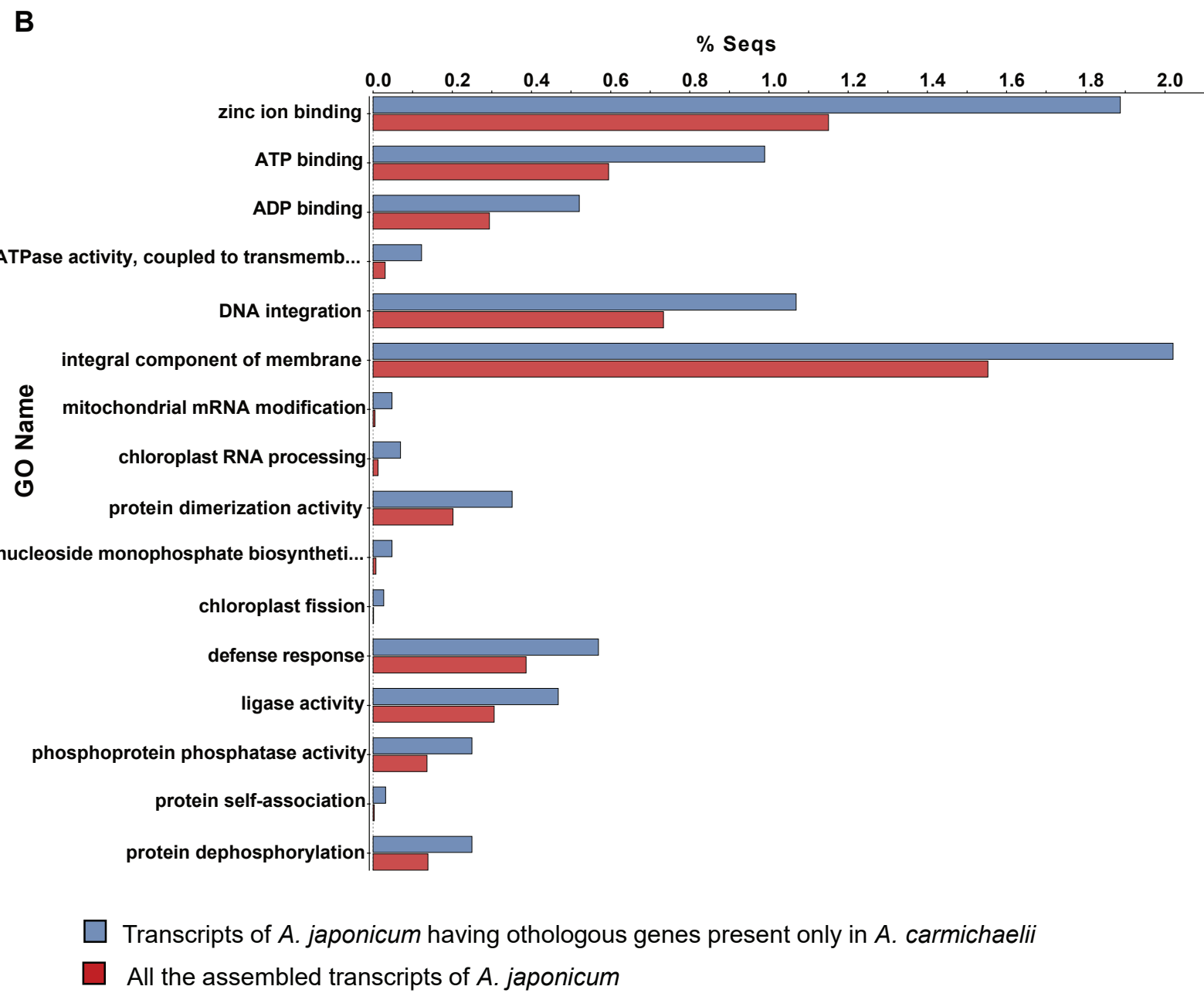

Supplement: Supplementary file 17 — Supplementary Figure 5: Comparative transcriptome analysis of Aconitum japonicum with two other Aconitum species, including Aconitum carmichaelii and Aconitum heterophyllum. (A) Venn diagram representing the distribution of orthogroups in A. japonicum, A. carmichaelii, and A. heterophyllum (B) GO enrichment analysis for transcripts specific to Aconitum japonicum and Aconitum carmichaelii. Using transcripts of A. japonicum having orthologous genes present only in A. carmichaelii as a test set and all the transcripts of A. japonicum with GO annotation as a reference set, gene ontology enrichment analysis was performed using Fisher’s exact test with the p value cut-off set as < 0.05 (PDF 634 KB) [file 11418_2025_1881_MOESM17_ESM.pdf]

Supplementary Figure 6

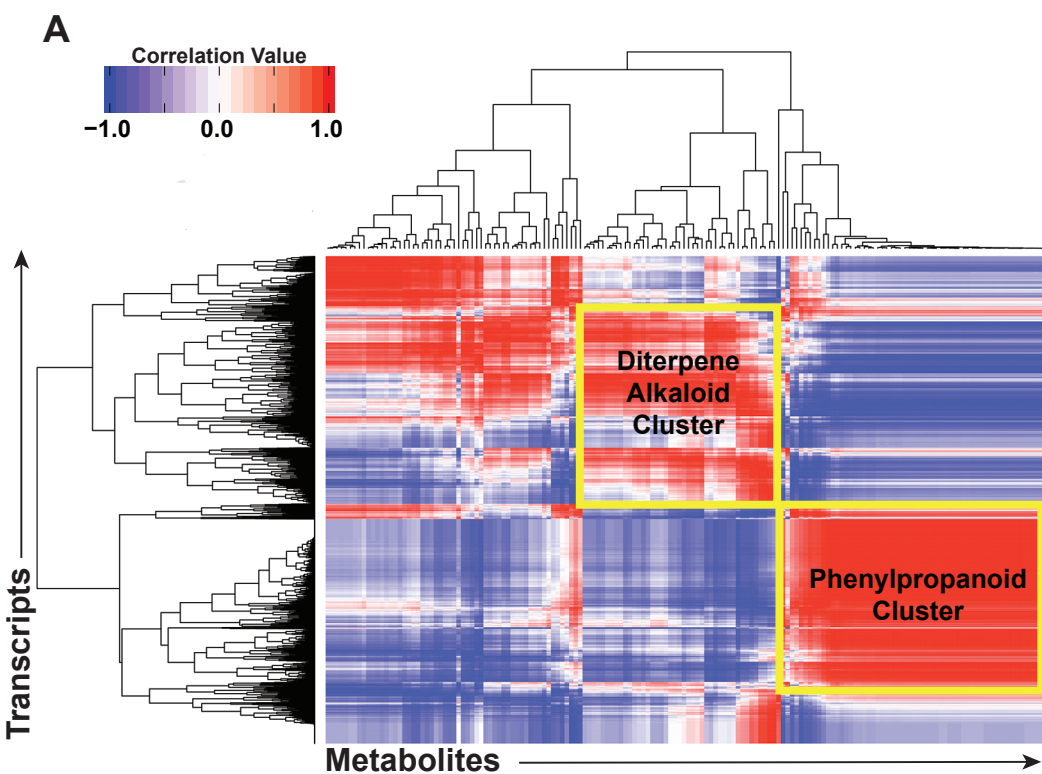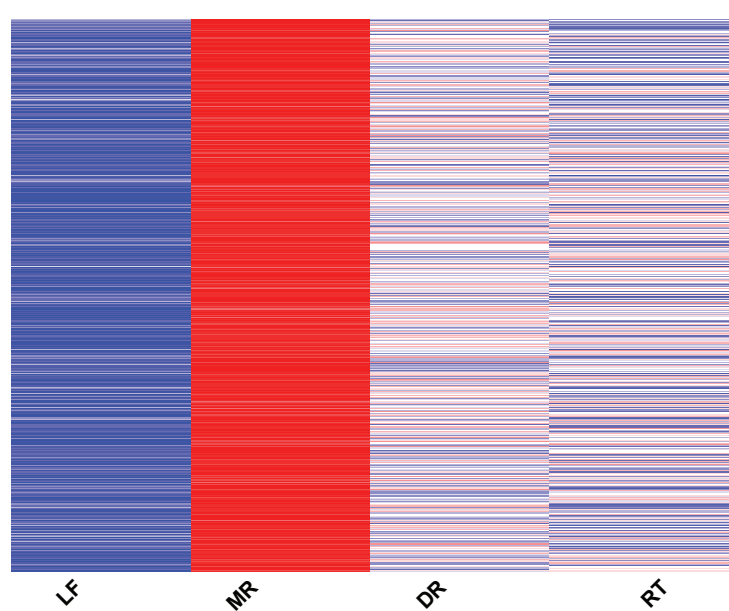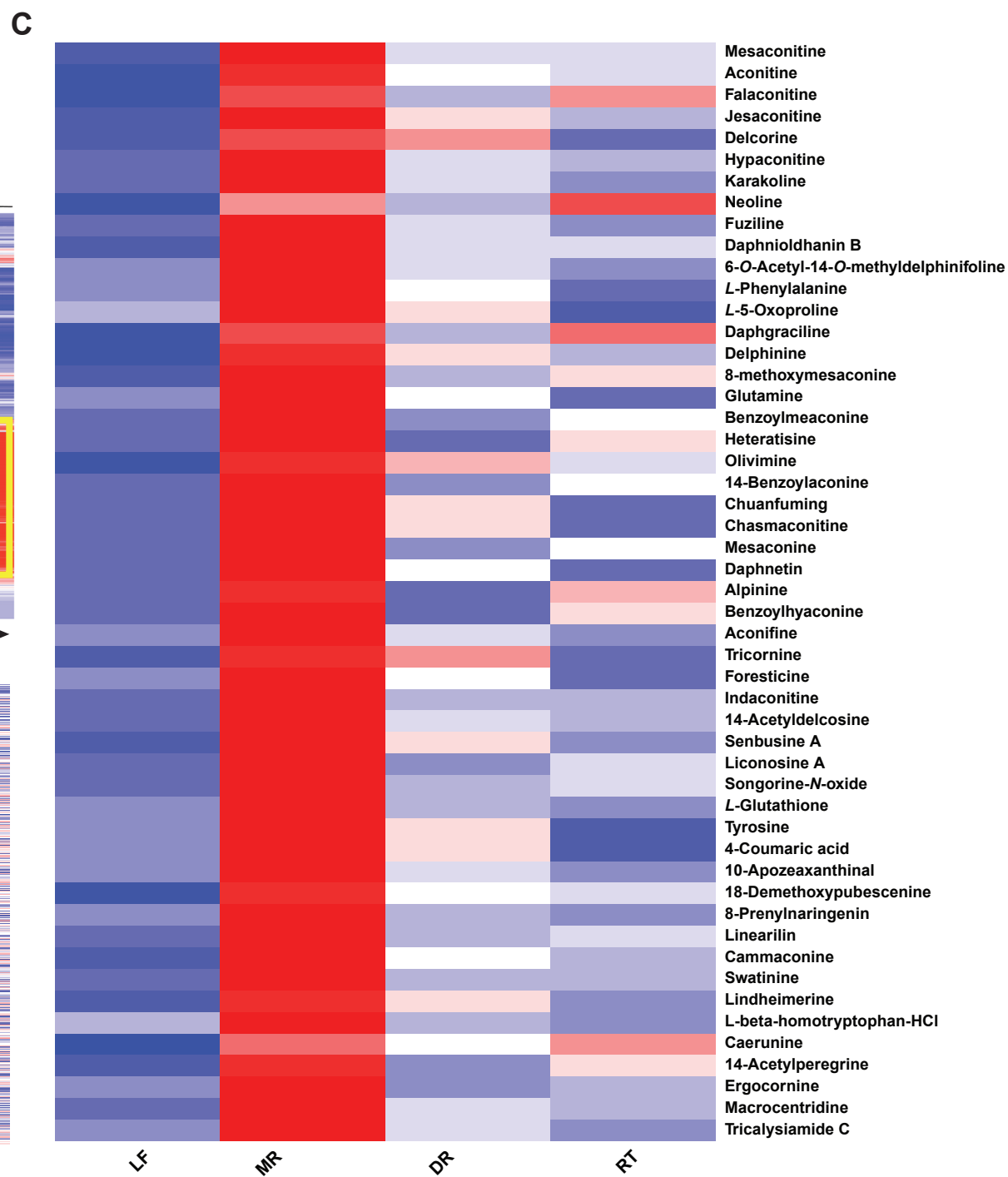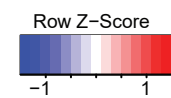

Supplement: Supplementary file 18 — Supplementary Figure 6: Correlation based integration analysis of the metabolome and transcriptome datasets of Aconitum japonicum. (A) Correlation coefficients were calculated between highly expressed annotated transcripts (FPKM>5) and the MS/MS-validated metabolites, and correlation scores are plotted as a heatmap with metabolites and transcripts represented along the X- and Y-axes, respectively. (B) The expression value of the transcripts included in the highly correlated cluster. The transcript name and expression data are included as the Supplementary Table 11. (C) The accumulation of the diterpene alkaloids included in the highly correlated cluster. Abbreviations: LF (leaf), MR (mother root), DR (daughter root), RT (rootlet) (PDF 3446 KB) [file 11418_2025_1881_MOESM18_ESM.pdf]
