# Supplementary material for: Multi-omics analysis reveals tissue-specific biosynthesis and accumulation of diterpene alkaloids in Aconitum japonicum
Source: J Nat Med. 2025 Mar 20;79(3):499–516. doi: 10.1007/s11418-025-01881-y (PMC12058934; doi:10.1007/s11418-025-01881-y)

Supplementary Figure 3

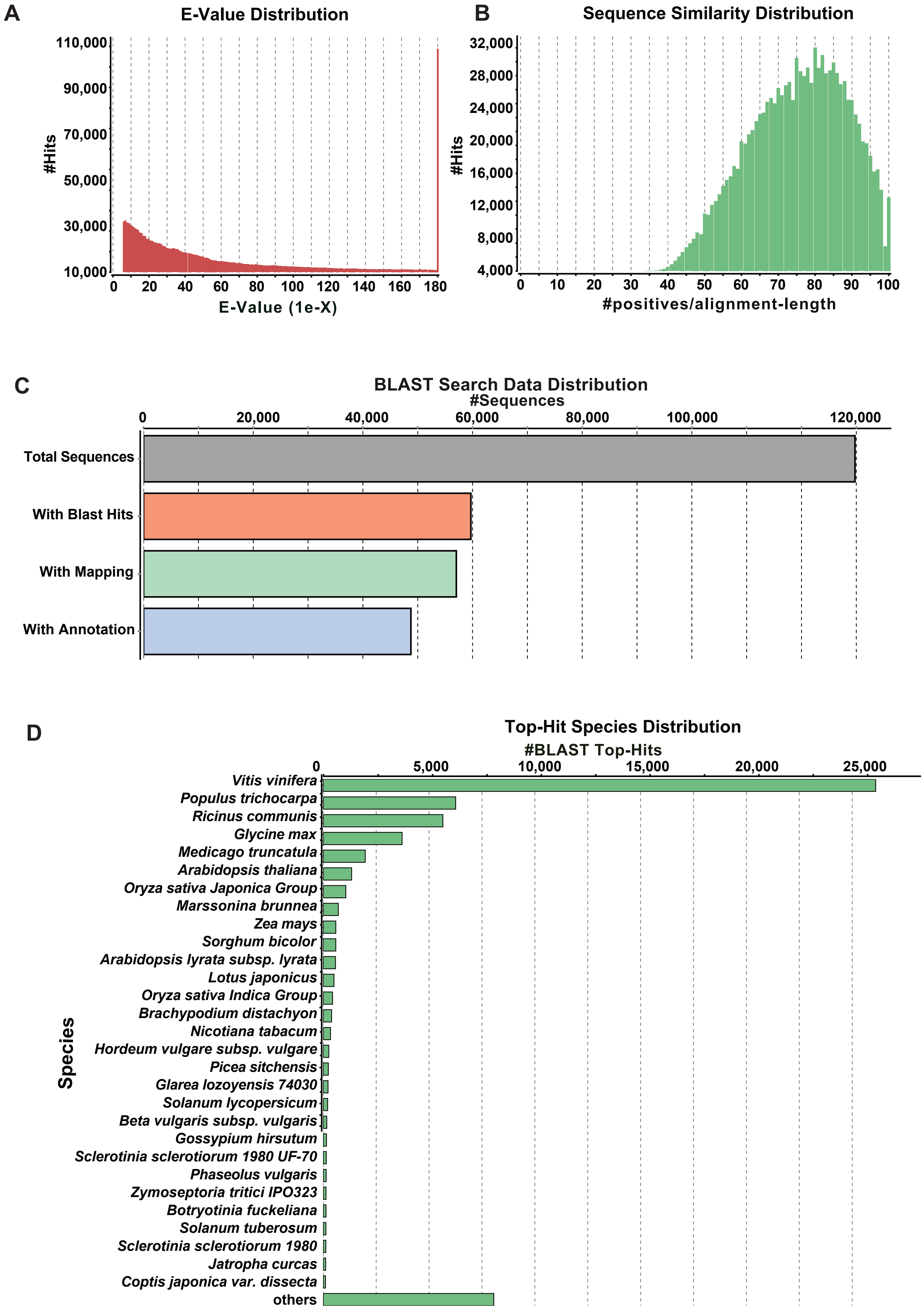

Supplement: Supplementary file 15 — Supplementary Figure 3: Blastx-based functional classification of the transcriptome assembly of Aconitum japonicum. (A) E-value distribution plot based on the Blast hits for the A. japonicum assembled transcriptome. (B) Sequence-similarity score distribution plot using top Blast hits used for the annotation of the de novo transcriptome assembly. (C) Bar chart representing data distribution of blast search-based annotation and Gene ontology assignment for A. japonicum transcriptome assembly using Blast2GO analysis. (D) Species distribution plot based on the top Blast hits for the assembled transcripts (PDF 506 KB) [file 11418_2025_1881_MOESM15_ESM.pdf]
